# Red Blood Cell Aggregation-Associated Dietary Pattern Predicts Hyperlipidemia and Metabolic Syndrome

**DOI:** 10.3390/nu10081127

**Published:** 2018-08-20

**Authors:** Pei Lin, Chun-Chao Chang, Kuo-Ching Yuan, Hsing-Jung Yeh, Sheng-Uei Fang, Tiong Cheng, Kai-Tse Teng, Kuo-Ching Chao, Jui-Hsiang Tang, Wei-Yu Kao, Pao-Ying Lin, Ju-Shian Liu, Jung-Su Chang

**Affiliations:** 1 School of Nutrition and Health Sciences, College of Nutrition, Taipei Medical University, Taipei 11031, Taiwan; peilin.nhs@gmail.com; 2 Division of Gastroenterology and Hepatology, Department of Internal Medicine, Taipei Medical University Hospital, Taipei 11031, Taiwan; chunchao@tmu.edu.tw (C.-C.C.); yiew@ms10.hinet.net (H.-J.Y.); f3836@yahoo.com.tw (S.-U.F.); janetiong@tmu.edu.tw (T.C.); momotenshi100@gmail.com (K.-T.T.); chin3064@gmail.com (K.-C.C.); 151002@h.tmu.edu.tw (J.-H.T.); 121021@h.tmu.edu.tw (W.-Y.K.); linpaoying@gmail.com (P.-Y.L.); liu6032@tmu.edu.tw (J.-S.L.); 3 Division of Gastroenterology and Hepatology, Department of Internal Medicine, School of Medicine, College of Medicine, Taipei Medical University, Taipei 11031, Taiwan; 4 Department of Emergency and Critical Care Medicine, Taipei Medical University Hospital, Taipei 11031, Taiwan; traumayuan@gmail.com; 5 Graduate Institute of Metabolism and Obesity Sciences, College of Nutrition, Taipei Medical University, Taipei 11031, Taiwan; 6 Nutrition Research Center, Taipei Medical University Hospital, Taipei 11031, Taiwan; 7 Chinese Taipei Society for the Study of Obesity, CTSSO, Taipei 11031, Taiwan

**Keywords:** red blood cell aggregation, hepcidin, soluble (s) CD163, dietary pattern, dyslipidemia, metabolic syndrome

## Abstract

Red blood cell (RBC) aggregation and iron status are interrelated and strongly influenced by dietary factors, and their alterations pose a great risk of dyslipidemia and metabolic syndrome (MetS). Currently, RBC aggregation-related dietary patterns remain unclear. This study investigated the dietary patterns that were associated with RBC aggregation and their predictive effects on hyperlipidemia and MetS. Anthropometric and blood biochemical data and food frequency questionnaires were collected from 212 adults. Dietary patterns were derived using reduced rank regression from 32 food groups. Adjusted linear regression showed that hepcidin, soluble CD163, and serum transferrin saturation (%TS) independently predicted RBC aggregation (all *p* < 0.01). Age-, sex-, and log-transformed body mass index (BMI)-adjusted prevalence rate ratio (PRR) showed a significant positive correlation between RBC aggregation and hyperlipidemia (*p*-trend < 0.05). RBC aggregation and iron-related dietary pattern scores (high consumption of noodles and deep-fried foods and low intake of steamed, boiled, and raw food, dairy products, orange, red, and purple vegetables, white and light-green vegetables, seafood, and rice) were also significantly associated with hyperlipidemia (*p*-trend < 0.05) and MetS (*p*-trend = 0.01) after adjusting for age, sex, and log-transformed BMI. Our results may help dieticians develop dietary strategies for preventing dyslipidemia and MetS.

## 1. Introduction

Obesity is driven by the consumption of calorie-dense foods (e.g., deep-fried foods or high-fat diet (HFD)), together with inadequate physical activity. Dyslipidemia and metabolic syndrome (MetS) are two of the most common obesity-related non-communicable diseases [1]. Dyslipidemia is the abnormal amount of lipids in the blood [2]. It signifies a decrease in the concentration of high-density lipoprotein cholesterol (HDL-C) and an increase in the levels of triglycerides (TG), total cholesterol, low-density lipoprotein cholesterol (LDL-C), and in the ratio of total cholesterol (total C) to HDL-C [3]. Another term, hyperlipidemia, is also used to described the elevations of fasting total C and TG and abnormalities of lipoproteins [4]. MetS is a clustering of metabolic abnormalities, including visceral obesity, hypertension, insulin resistance, low HDL-C, and high TG [5]. These conditions are closely related to obesity or overnutrition.

One’s diet can alter red blood cell (RBC) functions by affecting the membrane components of RBCs [6,7], RBC membrane fluidity, and the rheological function of RBCs [8,9]. The production of RBCs initially occurs in the bone marrow, and a sufficient amount of iron plays a critical role in this process. Mature RBCs take on a biconcave shape with a diameter of approximately 8 μm, a thickness of around 2 μm, and a life span of 120 days. Their unique shape creates a large surface area for gas exchange, and their small size allows RBCs to enter microcapillaries in tissues with minimal damage [10]. Studies found that an HFD induces alterations in RBC membrane phospholipids [7], and such alterations in the membrane lipid profiles of RBCs trigger macrophage adhesion to endothelial cells [11]. An HFD tends to alter RBC rheology. According to Cicha, Suzuki, Tateishi, and Maeda [9], diets rich in saturated fats and cholesterol can influence the blood’s viscosity and RBC aggregability. A multivariate analysis showed that the TG level independently predicted the elongation index of RBCs [12].

Iron dysregulation may also lead to RBC dysfunction. Iron is an essential component for the synthesis of hemoglobin (Hb) [13], an oxygen transport protein. More than 95% of cytoplasmic proteins in RBCs are composed of Hb. Hb in circulating RBCs contains almost 66.7% of the body’s iron. Since iron is a well-known catalyst of lipid peroxidation, an iron overload can lead to RBC disruption through peroxidative damage [14]. However, an iron deficiency does not prevent this situation. Previous studies found that intracellular scavengers of free radicals, such as vitamin E and glutathione peroxidase, decrease during an iron deficiency [15,16]. Another study in 1983 indicated that a low Hb concentration in RBCs may provide a greater opportunity for free radicals to react with cell membranes [17]. Yip et al. [17] also found in both rat and human studies, that an iron deficiency decreased RBC deformability due to the increase in membrane rigidity or a lack of sufficient membrane surfaces for full deformation of the cell. Nevertheless, the relationship between iron dysregulation and RBC aggregation in obese individuals remains unclear.

Studies showed that obesity is characterized by dysfunction of RBCs and iron metabolism [12,18]. RBCs and iron dysregulation are also strongly correlated with metabolic disorders [19,20,21], but the causal relationship between dysregulated iron metabolism and RBC dysfunction remains largely unknown. Obesity induces inflammation, causing an increase in circulating hepcidin and soluble cluster of differentiation 163 (sCD163) levels [18,22]. Hepcidin, a hormone produced by the liver, is the master iron regulator. It controls iron homeostasis by inhibiting the release of iron into the plasma through three mechanisms: (1) by inhibiting dietary iron absorption in the duodenum, (2) by regulating the release of recycled heme iron from CD163^+^ macrophages, and (3) by controlling the release of stored iron from hepatocytes or the spleen [23]. CD163 is a receptor located on macrophages which recycles heme iron from the haptoglobin (Hp)–Hb complex. When RBCs are lysed, free Hb is released and immediately binds to Hp in order to prevent oxidative damage from free Hb. The Hp–Hb complex is then taken up by macrophages through the receptor CD163 [24]. In specific conditions, when Hp is depleted, CD163 can directly pick up free Hb [25]. However, during inflammation, CD163 is cleaved into a soluble form by the proteolytic action of the metalloproteinase tumor necrosis factor (TNF)-α-converting enzyme (TACE/ADAM17) [26]. Therefore, circulating sCD163 is considered an inflammation marker and is frequently associated with obesity and metabolic disorders [26].

We hypothesized that obesity-related inflammation may cause the upregulation of hepcidin and sCD 163, and the altered serum hepcidin may affect iron levels, which, in turn, may influence RBC aggregation. The aims of this study were to: (1) investigate the relationship between serum iron biomarkers and RBC aggregation and (2) identify dietary patterns associated with RBC aggregation and their predictive effects on hyperlipidemia and MetS in 212 Taiwanese adults.

## 2. Materials and Methods 

### 2.1. Participants

In total, 212 Taiwanese adults aged 20–64 years were recruited at the Division of Gastroenterology and Hepatobiliary Diseases, Department of Internal Medicine, Taipei Medical University Hospital, from 29 April 2015 to 28 April 2016. A non-probability volunteering sampling was used as the sampling method. All participants were Han Chinese and were excluded from the study if they had at least one of the following: (1) were pregnant or breast feeding; (2) were taking hormone drugs; (3) had been diagnosed with hepatitis virus B or C or liver carcinoma; (4) failed to give a blood sample. This procedure was approved by the Taipei Medical University Institutional Review Board (TMU-JIRB 201502018), and written informed consent was signed by all participants.

### 2.2. Definitions

Hyperlipidemia was diagnosed in individuals with at least one of the following: (1) TG ≥ 200 mg/dL; (2) total C ≥ 240 mg/dL; (3) HDL-C < 40 mg/dL; (4) LDL-C ≥ 160 mg/dL; (5) a total C/HDL-C ratio ≥ 5 [27]. The criteria of MetS were based on the modified National Cholesterol Education Program Adult Treatment Panel III for the Asia Pacific [28]. Participants with at least three of the following were classified as having MetS: (1) a waist circumference ≥90 cm in males, and ≥80 cm in females (also defined as central obesity); (2) systolic blood pressure ≥130 mmHg or diastolic blood pressure ≥85 mmHg; (3) fasting blood glucose ≥100 mg/dL; (4) HDL-C < 40 mg/dL; (5) fasting TG ≥ 150 mg/dL.

### 2.3. Questionnaires

A simple questionnaire was used to record the basic information of participants, including age, sex, anthropometry, alcohol consumption, and history of diseases and medication. A food frequency questionnaire (FFQ) was used to investigate the dietary patterns of the participants. This was modified from a Chinese FFQ, which originally consisted of 64 items [29], and included three major categories: (1) food intake frequency; (2) cooking method used; (3) frequency of eating outside the home. The modified FFQ contained 66 food items that were categorized into 32 food groups, including five commonly used cooking methods for protein-rich foods and the frequencies of eating outside and homemade food. The food frequency was divided into eight levels: (1) 0–1 time/week; (2) 2–3 times/week; (3) 4–5 times/week; (4) 6–7 times/week; (5) 8–10 times/week; (6) 11–13 times/week; (7) 14–16 times/week; (8) ≥17 times/week.

### 2.4. Anthropometric Measurements

Body weight, height, waist and hip circumference of each participants were recorded, and the body mass index (BMI) was then calculated in kg/m^2^. The waist circumference was measured around the midpoint between the lower margin of the last rib and the top of the iliac crest.

### 2.5. Laboratory Measurements

Samples consisting of 15 mL of blood were collected after 8 h of fasting. Blood analyses included a complete blood cell count, inflammation biomarkers analysis, lipid profile, and serum iron biomarkers analysis. Serum iron biomarker analysis included serum ferritin (SF), serum iron, total iron-binding capacity (TIBC), serum-free Hb, serum hepcidin, and sCD163. Serum transferrin saturation (%TS) was calculated using the formula: (serum iron ÷ TIBC) × 100%. Serum iron was measured by a colorimetric method (Le-Zen Clinical Laboratory, Taipei, Taiwan). Serum-free Hb (Immunology Consultants Laboratory, Portland, OR, USA), serum hepcidin (DRG International Inc, Springfield, NJ, USA), and sCD163 (R&D Systems, Minneapolis, MN, USA) were analyzed by an enzyme-linked immunosorbent assay (ELISA) according to the manufacturer’s procedures. RBC aggregation was analyzed by pipetting 500 μL of whole blood into the left well of an RSD-K01 chip (MicroStar Instruments, Seoul, Korea) that was inserted into RheoScan-AnD300 (MicroStar Instruments, Seoul, Korea), a microfluidic ektacytometer, for measurement. The measurements provided the critical shear stress (CSS), which stands for the minimum amount of shear stress exerted by blood stream currents to disperse RBCs [30]. A greater value of CSS indicates higher RBC aggregability.

### 2.6. Statistical Analysis

**Figure 1:**
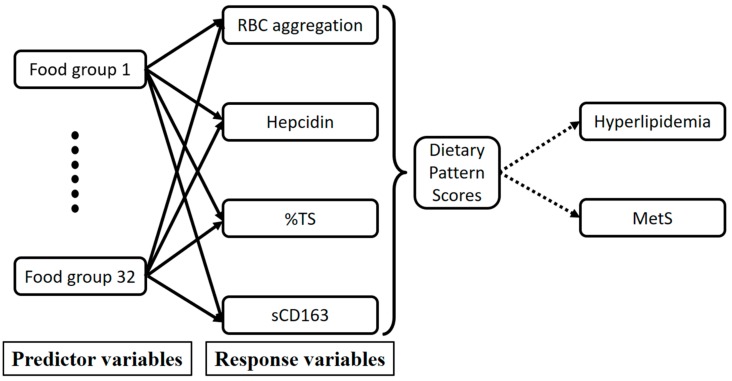
Directed acyclic graph of the reduced rank regression (RRR) conceptual framework. RBC: red blood cell; %TS: serum transferrin saturation; MetS: metabolic syndrome; sCD163: soluble cluster of differentiation 163.

Analyses were carried out using IBM^®^ SPSS^®^ 21 (IBM Corp., Armonk, NY, USA), SAS version 9.4 (SAS Institute Inc., Cary, NC, USA) and GraphPad Prism 5 (GraphPad Software, La Jolla, CA, USA). Categorical data were presented as the number (percentage (%)), and continuous data were presented as the mean ± standard deviation (SD). RBC aggregation data were divided into quartiles (Q) using SPSS by assigning Q1 to the smallest value. A general linear model was used to analyze the *p*-trend between variables for continuous data, and Chi-squared was used for categorical data. A normality test was carried out to test for the distribution of each variable. Variables that were not normally distributed were log-transformed. An age-, sex-, log-transformed BMI-adjusted Poisson regression model was performed to estimate the prevalence rate ratio (PRR) and 95% confidence interval (CI) of hyperlipidemia and MetS [31]. A multivariate linear regression analysis was implemented to examine the relationships between RBC aggregation and potential variables. A reduced rank regression (RRR) was carried out to derive RBC aggregation-associated dietary patterns with the 32 food groups from the FFQ as predictors, and biomarkers determined from the multivariate linear regression analysis as responses [32]. Food groups with factor loadings of ≥0.20 or ≤−0.20 were used to describe RBC aggregation-associated dietary patterns. The dietary pattern score was derived from each participant and represented the sum of food intake variables weighted by a factor loading. These scores indicated the conformity of food consumption to the RBC aggregation-associated dietary pattern. The directed acyclic graph below explains the conceptual framework of the RRR (Figure 1). If the *p*-values were ≤0.05, the differences were considered significant.

## 3. Results

The mean age and BMI of the study subjects were 41.94 ± 12.53 years (men: 41.60 ± 11.86 years, women: 42.27 ± 13.21 years; *p* = 0.705) and 24.56 ± 5.16 kg/m^2^ (men: 25.57 ± 4.20 kg/m^2^, women: 23.57 ± 5.81 kg/m^2^; *p* < 0.001), respectively. The prevalence rates of central obesity, hyperlipidemia, and MetS were 47.9% (men: 50.5%, women: 45.3%; *p* = 0.450), 38.2% (men: 39.0%, women: 37.4%; *p* = 0.803), and 25.5% (men: 36.7%, women: 24.3%; *p* = 0.692), respectively.

### 3.1. RBC Aggregation Shows a Positive Correlation with Dysregulated Iron and Is Positively Associated with Hyperlipidemia

We next stratified individuals according to the RBC aggregation concentrations. Table 1 shows that RBC aggregation was positively correlated with BMI (*p*-trend = 0.001), hyperlipidemia (*p*-trend < 0.001), MetS (*p*-trend = 0.001), blood lipids, and hepcidin (both *p*-trend < 0.01). On the other hand, %TS was a negatively correlated with RBC aggregation (*p*-trend = 0.001).

**Figure 2:**
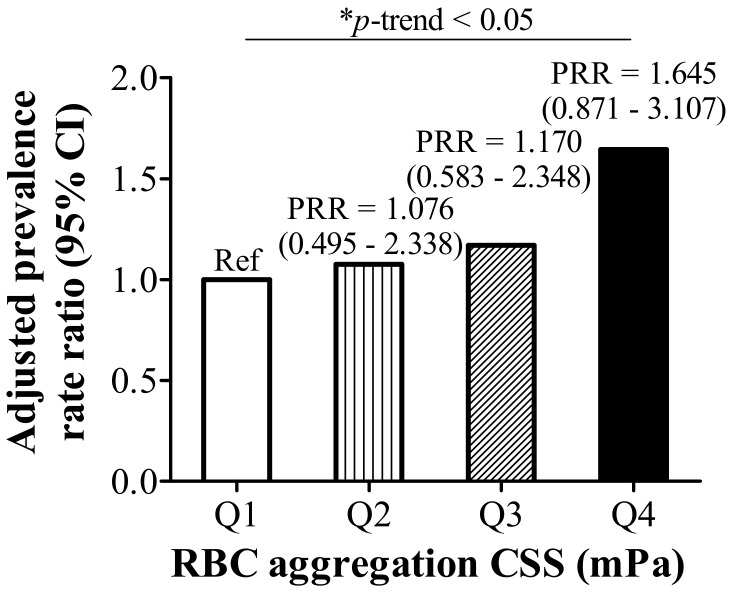
Prevalence rate ratio (PRR) and 95% confidence intervals of red blood cell (RBC) aggregation critical shear stress (CSS) quartile levels for hyperlipidemia adjusted by age, sex, and log-transformed body mass index (BMI); * *p* ≤ 0.05.

Age-, sex-, and log-transformed BMI-adjusted Poisson regression model showed that quartile levels of RBC aggregation CSS had a significant positive correlation with hyperlipidemia (*p*-trend < 0.05 (Figure 2)).

### 3.2. RBC Aggregation Is Positively Correlated with Hepcidin and sCD163, but Negatively Correlated with %TS

We next investigated potential confounding variables associated with RBC aggregation. A multivariate linear regression analysis was used to explore variables that could independently predict RBC aggregation. A univariate regression analysis indicated that log-transformed BMI (β = 0.316 (0.120–0.512), *p* < 0.01), hyperlipidemia (β = 0.169 (0.092–0.246), *p* < 0.001), MetS (β = 0.151 (0.065–0.237), *p* = 0.001), inflammation biomarkers (all *p* < 0.05), and lipid biomarkers (all *p* < 0.001, except for HDL-C) were positively correlated with RBC aggregation. For iron biomarkers, hepcidin (β = 0.0007 (0.0003–0.0010), *p* < 0.001) and log-transformed sCD163 (β = 0.152 (0.071–0.233), *p* < 0.001) were positively correlated with RBC aggregation, while %TS (β = −0.006 (−0.009–0.003), *p* < 0.001) was negatively correlated. After adjusting for covariates, only the iron biomarkers, which were hepcidin (β = 0.0009 (0.0005–0.0013), *p* < 0.001), log-transformed sCD163 (β = 0.116 (0.040–0.193), *p* < 0.01), and %TS (β = −0.006 (−0.010–0.003), *p* < 0.001), remained significantly correlated with RBC aggregation (Table 2; model 2).

**Figure 3:**
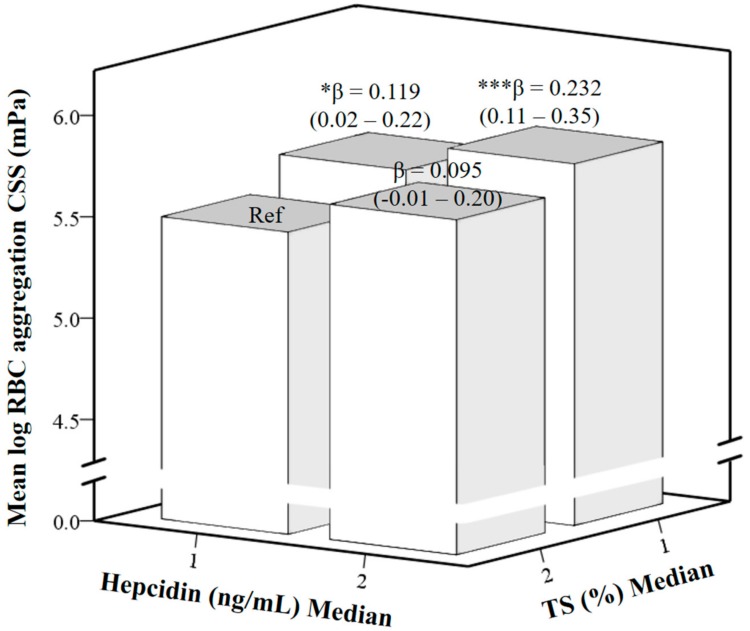
Multivariate linear regression of correlation between log-transformed red blood cell (RBC) aggregation critical shear stress (CSS), medians of hepcidin and transferrin saturation (%TS) levels adjusted by age, sex, and log-transformed body mass index (BMI); β, unstandardized coefficients. * *p* ≤ 0.05, *** *p* ≤ 0.001.

A further investigation was performed to investigate the dual effect of hepcidin and %TS on RBC aggregation by using multivariate linear regression analysis (Figure 3). Hepcidin and %TS were divided into median (M) groups, with M1 as the lower level and M2 as the higher level. A reference (Ref) was set in hepcidin M1 and %TS M2 groups. The participants in this group were considered to have sufficient iron levels compared to other groups due to lower levels of hepcidin and higher levels of %TS. When hepcidin remained at the M1 level but %TS decreased to the M1 level, RBC aggregation significantly increased (β = 0.119 (0.02–0.22), *p* < 0.05) compared to the Ref. When hepcidin increased to the M2 level and %TS decreased to the M1 level, RBC aggregation increased to an even larger extent (β = 0.232 (0.11–0.35), *p* < 0.001).

### 3.3. RBC Aggregation-Associated Dietary Patterns Independently Predict Hyperlipidemia and MetS

RBC aggregation-associated dietary pattern scores were derived by the RRR. The response variables were selected on the basis of strong correlations between the independent variables, which were hepcidin (*p* < 0.001), log-transformed sCD163 (*p* < 0.01) and %TS (*p* < 0.001), and RBC aggregation (Table 2; model 2). Table 3 shows the percentage of food variation corresponding to the first dietary pattern scores and factor loading of the food groups. Noodles and deep-fried foods were positively correlated with the first dietary pattern scores (factor loadings ≥0.20). On the other hand, steamed, boiled, and raw foods, dairy products, orange, red, and purple vegetables, white and light-green vegetables, seafood, and rice were negatively correlated with the dietary pattern scores (factor loadings ≤−0.20).

Table 4 shows a positive correlation between RBC aggregation-related dietary pattern scores and RBC aggregation after adjusting for age, sex, log BMI, and hyperlipidemia (*p*-trend = 0.01, model 5).

**Figure 4:**
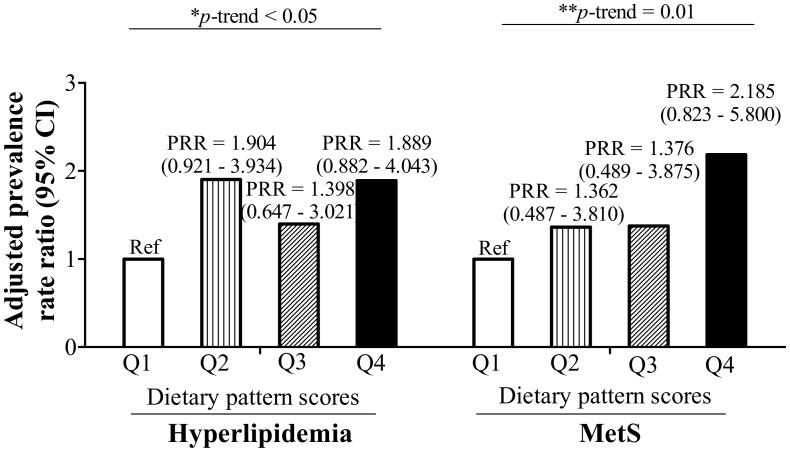
Prevalence rate ratio (PRR) and 95% confidence intervals of dietary pattern score quartile levels for hyperlipidemia and metabolic syndrome (MetS) adjusted for age, sex, and log-transformed body mass index (BMI). * *p* ≤ 0.05, ** *p* ≤ 0.01.

Age-, sex-, and log-transformed BMI-adjusted Poisson regression model showed that the quartile levels of dietary pattern scores had a significant positive correlation with hyperlipidemia (*p*-trend < 0.05 (Figure 4)) and MetS (*p*-trend = 0.01 (Figure 4)).

## 4. Discussion

Our study found that RBC aggregation is closely linked to obesity and dysregulated iron metabolism (as indicated by decreased %TS and increased hepcidin and sCD163). Increased RBC aggregation also increased the rate of hyperlipidemia (*p*-trend < 0.05). The RBC aggregation and iron-associated dietary pattern, which was characterized by high-frequency consumption of noodles and deep-fried foods and low-frequency intake of steamed, boiled, and raw food, dairy products, orange, red, and purple vegetables, white and light-green vegetables, seafood, and rice, was also significantly associated with both hyperlipidemia (*p*-trend < 0.05) and MetS (*p*-trend = 0.01).

One of the interesting findings of our results was the relationship between RBC aggregation and the consumption frequency of staple foods. Our study showed that noodles, but not rice, had the highest impact on the RBC aggregation and iron-associated dietary patterns (highest percentage of explained variation, 12.66%, and highest factor loading, 0.38). Both noodles and rice are refined carbohydrates which also yield a high glycemic index [33]. A recent study from the Chinese Nutrition and Health survey showed that, compared to rice, increased consumption of noodles produced a higher risk of developing type 2 diabetes [34]. Another study also showed negative effects of noodles on serum lipid levels and glucose metabolism [35]. Our dietary patterns showed that an increased intake of noodles was associated with deep-fried food consumption but decreased consumption of rice, steamed, boiled, and raw food, dairy products, orange, red, and purple vegetables, white and light-green vegetables, and seafood. This suggests that individuals who prefer noodles as a staple food are also less likely to eat steamed, boiled, and raw foods, vegetables, and seafood. Several prospective studies have suggested that a low intake of dairy products and vegetables increases the risks of dyslipidemia and MetS [35,36]. Our results also agree with previous studies indicating that deep-fried foods increase RBC aggregability, dyslipidemia, and MetS [36].

In this study, RBC aggregation and the three independent RBC aggregation factors, i.e., the iron biomarkers hepcidin, %TS, and sCD163, were selected as responses. The selected iron biomarkers had different relationships with RBC aggregation. Using Pearson correlation coefficients (data not shown), the *r* values of RBC aggregation and hepcidin, %TS, and sCD163 were 0.262 (*p* < 0.001), −0.254 (*p* < 0.001), and 0.285 (*p* < 0.001), respectively. Also, hepcidin was significantly correlated with %TS (r = 0.258, *p* < 0.001). However, correlations of sCD163 with hepcidin and %TS were not statistically significant. We further analyzed the relationship between the selected responses (RBC aggregation, hepcidin, %TS, and sCD163) and the selected food groups to clarify which kind of foods affected each response (data not shown). Using a linear regression, only steamed, boiled, and raw foods were negatively correlated with RBC aggregation (β: −0.030 (−0.056–0.003), *p* < 0.05). Hepcidin was positively correlated with deep-fried foods (β: 23.508 (3.717–43.298), *p* < 0.05), and sCD163 was negatively correlated with white and light-green vegetables (β: −0.064 (−0.125–0.003), *p* < 0.05). Noodles and steamed, boiled, and raw foods may also affect sCD163 as their *p*-value were close to significance (both *p* = 0.51). The %TS was not statistically significantly correlated with any of the selected food groups. Taken together, our data suggests that RBC aggregation-associated dietary pattern is largely influenced by RBC aggregation or the iron biomarkers hepcidin and sCD163.

Another interesting finding was that, although there was a statistically significant *p*-trend for the relationship between dietary pattern scores and hyperlipidemia (*p*-trend < 0.05), the PRRs between dietary pattern scores and hyperlipidemia did not consistently increase. Individuals with dietary pattern scores Q2 (PRR = 1.904 (0.921–3.934)) and Q4 (PRR = 1.889 (0.882–4.043)) had a higher risk of developing hyperlipidemia compared to Q1 (Figure 4). From the analysis between dietary pattern scores and variables (data not shown), only total C exhibited a significant difference between dietary pattern scores Q2 and Q3 (*p* < 0.05). Since total C was significantly higher in dietary pattern scores Q2 (208.38 ± 40.86) than in Q3 (193.57 ± 37.66), and one of the criteria for hyperlipidemia is elevated total C, high total C could be a possible reason for the Q2 dietary pattern scores having a higher rate of developing hyperlipidemia than Q3. Another possible explanation could be due to the gender element. Studies already showed that there are gender differences in eating behaviors [37,38,39]. Therefore, the phenomenon of Q2 having higher a risk of developing hyperlipidemia than Q3 may be due to the bias caused by different food preferences between sexes.

In the current study, hepcidin, %TS, and sCD163 were identified as independent factors which predicted RBC aggregation, with hepcidin and log-transformed sCD163 being positively correlated, and %TS being negatively correlated. However, the pathways through which iron biomarkers regulate RBC aggregation are still unknown. Hepcidin is regulated through a complex interplay of signals, mainly inflammation, the iron status, and RBC production. During obesity-induced inflammation, anemia of inflammation (AI) may occur as hepcidin increases. Inflammatory cytokines, predominately interleukin (IL)-6, promote the secretion of hepcidin through the Janus kinase (JAK)/signal transducer and activator of transcription 3 (STAT3) pathway. Therefore, the characteristics of AI include a decreased availability of circulating iron for the production of RBCs, despite adequate iron stores [40].

CD163 is the macrophage scavenger receptor which takes up Hp–Hb complexes, but sCD163 levels increase with obesity and metabolic disorders. Studies showed that the shedding of CD163 from macrophages during chronic inflammation is more robust than during acute inflammation [41]. Increases in sCD163 under inflammatory conditions are due to activation of TACE/ADAM17 [42]. TACE/ADAM17 is located in cell membranes and quickly responds to various physiological stimuli. Activation of the enzyme can be stimulated by Toll-like receptor (TLR) [43], which is activated by lipopolysaccharides [44], cross-linking oxidative stress [45], and thrombin [46]. Several studies indicated that sCD163 is a predictor of obesity-related diseases. sCD163 has strong correlations with MetS and inflammatory serum markers [22]. CD163^+^ macrophages can be found in vessel walls, and increased expressions of heme oxygenase (HO)-1 and CD163 were positively correlated with tissue iron content and symptomatic plaques [47]. However, how sCD163 affects RBCs and RBC aggregability is still unclear.

This study has several limitations. First, the sample size was relatively small. Second, the FFQ only represents the frequency of participants’ food intake, while the actual amount of food consumed by participants remains unknown. Thus, the precise amounts of nutrients could not be determined. Third, the RRR is a method that requires in-depth knowledge of the relationships between diet and disease in order to select the response variables. Using different variables for the RRR analysis will yield different results. Since different variables have different relationships with each other and with the food groups, choosing different biomarkers as response variables will affect the outcome of the RRR analysis.

## 5. Conclusions

The study results suggest that individuals with the highest consumption frequencies of noodles and deep-fried foods and the lowest intake of steamed, boiled, and raw food, dairy products, orange, red, and purple vegetables, white and light-green vegetables, seafood, and rice were more likely to develop RBC aggregation, dyslipidemia, and MetS. Our findings may help clinicians and dieticians develop dietary strategies for preventing dyslipidemia and MetS particularly in those individuals with RBC and iron dysfunctions.

## Figures and Tables

**Table 1:** Baseline characteristics of the study population according to quartiles of RBC aggregation levels (*N* = 196).

	RBC Aggregation CSS (mPa), Quartiles ^$^				*p*-Trend
	Q1 (*n* = 48)	Q2 (*n* = 49)	Q3 (*n* = 50)	Q4 (*n* = 49)	
Age (years)	42.13 ± 13.99	40.08 ± 13.00	41.51 ± 11.53	46.63 ± 10.68	0.055
BMI (kg/m^2^)	23.72 ± 4.97	23.12 ± 4.02	24.86 ± 5.61	27.16 ± 5.64	0.001
Male (n, %)	23 (47.9)	24 (49.0)	24 (49.8)	24 (49.0)	0.999
Hyperlipidemia (n, %)	15 (31.3)	12 (24.5)	18 (36.0)	31 (63.3)	<0.001
MetS (n, %)	11 (22.9)	5 (10.2)	15 (30.0)	22 (44.9)	0.001
**Lipids**					
Total C (mg/dL)	188.44 ± 38.73	193.00 ± 27.01	200.60 ± 36.92	213.49 ± 41.69	0.005
TG (mg/dL)	99.54 ± 67.29	100.29 ± 63.02	116.18 ± 67.40	165.88 ± 88.67	<0.001
HDL-C (mg/dL)	60.21 ± 15.60	56.33 ± 12.59	55.10 ± 16.48	53.73 ± 15.78	0.184
LDL-C (mg/dL)	105.83 ± 30.81	115.31 ± 25.52	120.94 ± 31.23	129.24 ± 35.53	0.003
**Iron**					
HCT (%)	42.28 ± 5.58	43.72 ± 7.13	42.33 ± 7.97	43.90 ± 8.90	0.577
Hb (g/dL)	14.55 ± 1.99	15.00 ± 2.63	14.44 ± 3.05	15.04 ± 3.18	0.614
Free Hb (μg/mL)	157.27 ± 49.48	143.84 ± 52.73	162.09 ± 45.42	154.99 ± 59.97	0.472
SF (ng/mL)	141.74 ± 169.22	131.27 ± 103.73	139.56 ± 167.79	189.90 ± 137.88	0.191
TS (%)	31.51 ± 12.05	35.01 ± 12.21	27.97 ± 13.75	25.71 ± 8.67	0.001
Hepcidin (ng/mL)	116.87 ± 101.17	151.07 ± 86.61	136.78 ± 102.47	207.19 ± 123.12	<0.001
sCD163 (ng/mL)	761.47 ± 470.38	744.03 ± 411.93	810.59 ± 299.62	978.99 ± 514.13	0.069

*p*-trend values were analyzed by a general linear model for continuous variables, and Chi-squared for categorical variables. Continuous data are presented as the mean ± standard deviation, while categorical data are presented as a number (percentage of the same group). ^$^ Red blood cell (RBC) aggregation critical shear stress (CSS) quartiles: Quartile 1, male ≤ 224.35, female ≤ 239.03; Quartile 2, 224.35 < male ≤ 263.12, 239.03 < female ≤ 284.21; Quartile 3, 263.12 < male ≤ 324.55, 284.21 < female ≤ 351.37; Quartile 4, male > 324.55, female > 351.37 mPa. BMI: body mass index; C: cholesterol; TG: triglycerides; HDL-C: high-density lipoprotein cholesterol; LDL-C: low-density lipoprotein cholesterol; HCT: hematocrit; Hb: hemoglobin; SF: ferritin; TS: serum transferrin saturation; sCD163: soluble cluster of differentiation 163.

**Table 2:** Multivariate linear regression of correlations between log-transformed RBC aggregation and selected anthropometric, inflammation, lipid, glucose, and iron indicators.

	Univariate		Model 1 ^#^		Model 2 ^$^	
	ß (95% CI)	*p*-Value	ß (95% CI)	*p*-Value	ß (95% CI)	*p*-Value
Age (years)	0.004 (0.001–0.007)	0.020	0.003 (0.000–0.006)	0.035	0.001 (−0.003–0.004)	0.713
Log BMI (kg/m^2^)	0.316 (0.120–0.512)	0.002	0.378 (0.182–0.574)	<0.001	0.010 (−0.195–0.215)	0.923
Hyperlipidemia						
Control	Ref		Ref		Ref	
Hyperlipidemia	0.169 (0.092–0.246)	<0.001	0.124 (0.042–0.205)	0.003	0.025 (−0.061–0.110)	0.572
MetS						
Control	Ref		Ref			
MetS	0.151 (0.065–0.237)	0.001	0.072 (−0.027–0.171)	0.155		
**Lipids**						
Log total C (mg/dL)	0.450 (0.247–0.653)	<0.001	0.390 (0.192–0.587)	<0.001		
Log TG (mg/dL)	0.144 (0.084–0.203)	<0.001	0.131 (0.061–0.201)	<0.001		
Log HDL-C (mg/dL)	−0.102 (−0.254–0.050)	0.188				
LDL-C (mg/dL)	0.002 (0.001–0.004)	<0.001	0.002 (0.001–0.003)	<0.001	0.001 (0.000–0.002)	0.073
**Iron**						
Log HCT (%)	−0.087 (−0.324–0.151)	0.472				
Log Hb (g/dL)	−0.104 (−0.322–0.114)	0.350				
Free Hb (μg/mL)	0.000 (−0.001–0.001)	0.896				
Log SF (ng/mL)	0.021 (−0.012–0.053)	0.208				
TS (%)	−0.006 (−0.009–0.003)	<0.001	−0.004 (−0.007–0.001)	0.017	−0.006 (−0.010–0.003)	<0.001
Hepcidin (ng/mL)	0.0007 (0.0003–0.0010)	<0.001	0.0008 (0.0004–0.0011)	<0.001	0.0009 (0.0005–0.0013)	<0.001
Log sCD163 (ng/mL)	0.152 (0.071–0.233)	<0.001	0.119 (0.037–0.201)	0.005	0.116 (0.040–0.193)	0.003

^#^ Model 1: Adjusted for age, sex, and log BMI; ^$^ Model 2: Adjusted for age, sex, log BMI, LDL-C, TS, hepcidin, and log sCD163. RBC: red blood cell; BMI: body mass index; MetS: metabolic syndrome; C: cholesterol; TG: triglycerides; HDL-C: high-density lipoprotein cholesterol; LDL-C: low-density lipoprotein cholesterol; HCT: hematocrit; Hb: hemoglobin; SF: ferritin; TS: serum transferrin saturation; sCD163: soluble cluster of differentiation 163.

**Table 3:** Food groups which were strongly associated with RBC aggregation-related dietary pattern scores identified by using an RRR.

Food Group	Explained Variation (%)	Factor Loading *
Noodles	12.66	0.38
Deep-fried foods	6.78	0.28
Steamed/boiled/raw foods	10.43	−0.34
Dairy products	7.73	−0.30
Orange/red/purple vegetables	7.49	−0.29
White/light-green vegetables	5.39	−0.25
Seafood	4.13	−0.22
Rice	3.74	−0.21
Total explained variation (%):	58.37	

* Factor loadings are correlations between food groups and the first dietary pattern scores (correlation coefficient for the RRR-derived pattern ≥ |0.20|). RRR: reduced rank regression.

**Table 4:** Adjusted linear regression of the relationship between the quartiles of dietary pattern score levels and log-transformed RBC aggregation.

	Dietary Pattern Scores							*p*-Trend
	Quartile 1	Quartile 2	*p*-Value	Quartile 3	*p*-Value	Quartile 4	*p*-Value	
Univariate	Ref	0.086 (−0.009–0.181)	0.076	0.086 (−0.016–0.189)	0.097	0.193 (0.084–0.302)	0.001	0.001
Model 1 *	Ref	0.083 (−0.011–0.177)	0.081	0.085 (−0.017–0.188)	0.101	0.180 (0.071–0.288)	0.001	0.002
Model 2 ^#^	Ref	0.087 (−0.007–0.180)	0.068	0.087 (−0.015–0.188)	0.093	0.208 (0.102–0.314)	<0.001	<0.001
Model 3 ^$^	Ref	0.085 (−0.004–0.174)	0.062	0.062 (−0.036–0.161)	0.214	0.190 (0.074–0.306)	0.002	0.010
Model 4 ^^^	Ref	0.065 (−0.028–0.158)	0.167	0.068 (−0.032–0.168)	0.178	0.155 (0.049–0.261)	0.005	0.004
Model 5 ^&^	Ref	0.069 (−0.021–0.159)	0.131	0.049 (−0.049–0.146)	0.322	0.158 (0.045–0.270)	0.007	0.024

* Model 1. adjusted for age; ^#^ Model 2: adjusted for age and sex; ^$^ Model 3: adjusted for age, sex, and log BMI; ^^^ Model 4: adjusted for age, sex, and hyperlipidemia; ^&^ Model 5: adjusted for age, sex, log BMI, and hyperlipidemia. RBC: red blood cell.

## References

[1] Misra A., Shrivastava U. (2013). Obesity and dyslipidemia in south Asians. Nutrients.

[2] Smith G. (2007). Epidemiology of dyslipidemia and economic burden on the healthcare system. Am. J. Manag. Care.

[3] Chang H.-Y., Yeh W.-T., Chang Y.-H., Tsai K.-S., Pan W.-H. (2002). Prevalence of dyslipidemia and mean blood lipid values in taiwan: Results from the nutrition and health survey in Taiwan (NAHSIT, 1993–1996). Chin. J. Physiol..

[4] Nelson R.H. (2013). Hyperlipidemia as a risk factor for cardiovascular disease. Prim. Care.

[5] Hwang L.-C., Bai C.-H., Chen C.-J. (2006). Prevalence of obesity and metabolic syndrome in Taiwan. J. Formos. Med. Assoc..

[6] Popp-Snijders C., Schouten J., Van Blitterswijk W., Van der Veen E. (1986). Changes in membrane lipid composition of human erythrocytes after dietary supplementation of (n-3) polyunsaturated fatty acids. Maintenance of membrane fluidity. Biochim. Biophys Acta-Biomembr..

[7] Novgorodtseva T.P., Karaman Y.K., Zhukova N.V. (2010). The effect of high fat food on erythrocyte phospholipids, fatty acids composition and glutathione redox-system of rats with alimentary dyslipidemia. Health.

[8] Cicha I., Suzuki Y., Tateishi N., Maeda N. (2001). Enhancement of red blood cell aggregation by plasma triglycerides. Clin. Hemorheol. Microcirc..

[9] Cicha I., Suzuki Y., Tateishi N., Maeda N. (2004). Effects of dietary triglycerides on rheological properties of human red blood cells. Clin. Hemorheol. Microcirc..

[10] Baskurt O.K., Meiselman H.J. (2003). Seminars in thrombosis and hemostasis. Blood Rheology and Hemodynamics.

[11] Unruh D., Srinivasan R., Benson T., Haigh S., Coyle D., Batra N., Keil R., Sturm R., Blanco V., Palascak M. (2015). Red blood cell dysfunction induced by high-fat diet: Potential implications for obesity-related atherosclerosis. Circulation.

[12] Wiewiora M., Piecuch J., Glûck M., Slowinska-Lozynska L., Sosada K. (2014). The impacts of super obesity versus morbid obesity on red blood cell aggregation and deformability among patients qualified for bariatric surgery. Clin. Hemorheol. Microcirc..

[13] Adamson J.W. (1994). The relationship of erythropoietin and iron metabolism to red blood cell production in humans. Semin. Oncol..

[14] Smith K.A., Mengel C.E. (1968). Association of iron-dextran-induced hemolysis and lipid peroxidation in mice. J. Lab. Clin. Med..

[15] Cellerino R., Guidi G., Perona G. (1976). Plasma iron and erythrocytic glutathione peroxidase activity. Eur. J. Haematol..

[16] Macdougall L.G. (1972). Red cell metabolism in iron deficiency anemia: III. The relationship between glutathione peroxidase, catalase, serum vitamin e, and susceptibility of iron-deficient red cells to oxidative hemolysis. J. Pediatr..

[17] Yip R., Mohandas N., Clark M.R., Jain S., Shohet S.B., Dallman P.R. (1983). Red cell membrane stiffness in iron deficiency. Blood.

[18] Tussing-Humphreys L., Pustacioglu C., Nemeth E., Braunschweig C. (2012). Rethinking iron regulation and assessment in iron deficiency, anemia of chronic disease, and obesity: Introducing hepcidin. J. Acad. Nutr. Diet..

[19] Huang L.L., Dou D.-M., Liu N., Wang X.X., Fu L.-Y., Wu X., Wang P. (2018). Association of erythrocyte parameters with metabolic syndrome in the pearl river delta region of china: A cross sectional study. BMJ Open.

[20] Kuhn V., Diederich L., Keller T.C.S., Kramer C.M., Lückstädt W., Panknin C., Suvorava T., Isakson B.E., Kelm M., Cortese-Krott M.M. (2017). Red blood cell function and dysfunction: Redox regulation, nitric oxide metabolism, anemia. Antioxid. Redox Signal..

[21] Datz C., Felder T.K., Niederseer D., Aigner E. (2013). Iron homeostasis in the metabolic syndrome. Eur. J. Clin. Investig..

[22] Møller H.J., Frikke-Schmidt R., Moestrup S.K., Nordestgaard B.G., Tybjærg-Hansen A. (2011). Serum soluble CD163 predicts risk of type 2 diabetes in the general population. Clin. Chem..

[23] Ganz T., Nemeth E. (2012). Hepcidin and iron homeostasis. Biochim. Biophys Acta-Mol. Cell Res..

[24] Nielsen M.J., Moestrup S.K. (2009). Receptor targeting of hemoglobin mediated by the haptoglobins: Roles beyond heme scavenging. Blood.

[25] Schaer D.J., Schaer C.A., Buehler P.W., Boykins R.A., Schoedon G., Alayash A.I., Schaffner A. (2006). CD163 is the macrophage scavenger receptor for native and chemically modified hemoglobins in the absence of haptoglobin. Blood.

[26] Parkner T., Sørensen L.P., Nielsen A.R., Fischer C.P., Bibby B.M., Nielsen S., Pedersen B.K., Møller H.J. (2012). Soluble CD163: A biomarker linking macrophages and insulin resistance. Diabetologia.

[27] Lin C.-F., Chang Y.-H., Chien S.-C., Lin Y.-H., Yeh H.-Y. (2018). Epidemiology of dyslipidemia in the Asia pacific region. Int. J. Gerontol..

[28] Tan C.-E., Ma S., Wai D., Chew S.-K., Tai E.-S. (2004). Can we apply the national cholesterol education program adult treatment panel definition of the metabolic syndrome to Asians?. Diabetes Care.

[29] Lee M.S., Pen W.H., Liu K.L., Yu M.S. (2006). Reproducibility and validity of a Chinese food frequency questionnaire used in Taiwan. Asia Pac. J. Clin. Nutr..

[30] Lee K., Priezzhev A., Shin S., Yaya F., Meglinski I. (2016). Characterization of shear stress preventing red blood cells aggregation at the individual cell level: The temperature dependence. Clin. Hemorheol. Microcirc..

[31] Barros A.J., Hirakata V.N. (2003). Alternatives for logistic regression in cross-sectional studies: An empirical comparison of models that directly estimate the prevalence ratio. BMC Med. Res. Methodol..

[32] Hoffmann K., Schulze M.B., Schienkiewitz A., Nöthlings U., Boeing H. (2004). Application of a new statistical method to derive dietary patterns in nutritional epidemiology. Am. J. Epidemiol..

[33] Atkinson F.S., Foster-Powell K., Brand-Miller J.C. (2008). International tables of glycemic index and glycemic load values: 2008. Diabetes Care.

[34] Batis C., Mendez M.A., Gordon-Larsen P., Sotres-Alvarez D., Adair L., Popkin B. (2016). Using both principal component analysis and reduced rank regression to study dietary patterns and diabetes in Chinese adults. Public Health Nutr..

[35] Song S.J., Lee J.E., Paik H.-Y., Park M.S., Song Y.J. (2012). Dietary patterns based on carbohydrate nutrition are associated with the risk for diabetes and dyslipidemia. Nutr. Res. Pract..

[36] Baxter A., Coyne T., McClintock C. (2006). Dietary patterns and metabolic syndrome-a review of epidemiologic evidence. Asia Pac. J. Clin. Nutr..

[37] Bédard A., Hudon A.-M., Drapeau V., Corneau L., Dodin S., Lemieux S. (2015). Gender differences in the appetite response to a satiating diet. J. Obes..

[38] Rolls B., Fedoroff I., Guthrie J. (1991). Gender differences in eating behavior and body weight regulation. Health Psychol..

[39] Wardle J., Haase A.M., Steptoe A., Nillapun M., Jonwutiwes K., Bellisie F. (2004). Gender differences in food choice: The contribution of health beliefs and dieting. Ann. Behav. Med..

[40] Wang C.-Y., Babitt J.L. (2016). Hepcidin regulation in the anemia of inflammation. Curr. Opin. Hematol..

[41] Zwadlo G., Voegeíi R., Osthoff K.S., Sorg C. (1987). A monoclonal antibody to a novel differentiation antigen on human macrophages associated with the down-regulatory phase of the inflammatory process. Pathobiology.

[42] Etzerodt A., Maniecki M.B., Møller K., Møller H.J., Moestrup S.K. (2010). Tumor necrosis factor α-converting enzyme (tace/adam17) mediates ectodomain shedding of the scavenger receptor CD163. J. Leukoc. Biol..

[43] Le Gall S.M., Maretzky T., Issuree P.D., Niu X.-D., Reiss K., Saftig P., Khokha R., Lundell D., Blobel C.P. (2010). Adam17 is regulated by a rapid and reversible mechanism that controls access to its catalytic site. J. Cell Sci..

[44] Hintz K.A., Rassias A.J., Wardwell K., Moss M.L., Morganelli P.M., Pioli P.A., Givan A.L., Wallace P.K., Yeager M.P., Guyre P.M. (2002). Endotoxin induces rapid metalloproteinase-mediated shedding followed by up-regulation of the monocyte hemoglobin scavenger receptor CD163. J. Leukoc. Biol..

[45] Timmermann M., Högger P. (2005). Oxidative stress and 8-iso-prostaglandin f2α induce ectodomain shedding of CD163 and release of tumor necrosis factor-α from human monocytes. Free Radic. Biol. Med..

[46] Chung S., Kim J.-E., Park S., Han K.-S., Kim H.K. (2011). Neutrophil and monocyte activation markers have prognostic impact in disseminated intravascular coagulation: In vitro effect of thrombin on monocyte CD163 shedding. Thromb. Res..

[47] Ijäs P., Nuotio K., Saksi J., Soinne L., Saimanen E., Karjalainen-Lindsberg M.-L., Salonen O., Sarna S., Tuimala J., Kovanen P.T. (2007). Microarray analysis reveals overexpression of CD163 and ho-1 in symptomatic carotid plaques. Arterioscler. Thromb. Vasc. Biol..

